# Evidence of Circulation and Phylogenetic Analysis of Hepatitis E Virus (HEV) in Wild Boar in South-East Italy

**DOI:** 10.3390/v15102021

**Published:** 2023-09-28

**Authors:** Gianfranco La Bella, Maria Grazia Basanisi, Gaia Nobili, Rosa Coppola, Annita Maria Damato, Adelia Donatiello, Gilda Occhiochiuso, Antonella Cristina Romano, Mariateresa Toce, Lucia Palazzo, Francesco Pellegrini, Angela Fanelli, Barbara Di Martino, Elisabetta Suffredini, Gianvito Lanave, Vito Martella, Giovanna La Salandra

**Affiliations:** 1 Istituto Zooprofilattico Sperimentale della Puglia e della Basilicata, 71121 Foggia, Italy; 2 Department of Veterinary Medicine, University of Bari Aldo Moro, 70010 Bari, Italy; 3 Department of Veterinary Medicine, Università degli Studi di Teramo, 64100 Teramo, Italy; 4 Department of Food Safety Nutrition and Veterinary Public Health, Istituto Superiore di Sanità, 00161 Rome, Italy

**Keywords:** hepatitis E virus, public health, whole genome, wild boar, zoonosis

## Abstract

Hepatitis E virus (HEV) is an important cause of acute viral hepatitis in humans worldwide. The food-borne transmission of HEV appears to be a major route in Europe through the consumption of pork and wild boar meat. HEV epidemiology in wild boars has been investigated mainly in Northern and Central Italian regions, whilst information from Southern Italy is limited. We investigated the occurrence of HEV in wild boar in the Apulia and Basilicata regions (Southern Italy). Thirteen (10.4%) out of one hundred and twenty-five wild boar samples tested positive for HEV using a quantitative reverse transcription PCR. HEV prevalence was 12% in Apulia and 9.3% in Basilicata. Seven samples were genotyped, and different subtypes (c, f, m) of genotype 3 were identified. The complete genome of a 3m strain was determined, and the virus showed the highest nucleotide identity to a human HEV strain identified in France in 2017. These findings demonstrate the substantial circulation of HEV in the wild boar population in Italian Southern regions. Gathering information on the HEV strains circulating in different geographical areas is useful for tracking the origin of HEV outbreaks and assessing the epidemiological role of wild boar as a potential virus reservoir for domestic pigs.

## 1. Introduction

Hepatitis E infection represents an important global health problem and is a significant cause of morbidity and mortality [1]. Each year, HEV infects an estimated 20 million people, leading to over 3.3 million clinically symptomatic cases, and the World Health Organization estimated approximately 44,000 deaths caused by hepatitis E in 2015 [2]. Clinical hepatitis E is generally self-limiting, although some cases may evolve into fulminant hepatitis, with possible fatal outcomes. In developed countries, chronic HEV infections are observed in immunosuppressed patients, notably in solid organ transplant recipients, in which rapid progression to cirrhosis is observed [3]. Extrahepatic symptoms, such as neurological, kidney or hematological dysfunctions, have also been described [4]. Between 2005 and 2015, more than 21,000 acute clinical cases with 28 associated fatalities were notified in EU countries, with an overall 10-fold increase in reported HEV cases [5]. The number of reported hepatitis E cases in Europe is underestimated, and data might not reflect the real number of infections in the EU. HEV infection is not mandatorily notified to public health authorities in all EU member states, and surveillance systems differ among countries. In Italy, the number of individuals infected with HEV rose over the 2007–2018 time span, doubling from 2018 to 2019. During 2021, 21 cases of infection were reported, with one death [6]. In Italy, viral hepatitis is a notifiable disease, although HEV is not yet included systematically in the diagnostic algorithms of hepatitis.

HEV is a hepatotropic, single-stranded positive-sense RNA virus with a genome of 6.4–7.2 kb, which is classified in the genus *Paslahepevirus* belonging to the family *Hepeviridae*, subfamily *Orthohepevirinae* [7], and can be further divided into two species: *Paslahepevirus balayani* and *Paslahepevirus alci*. The *Paslahepevirus balayani* species includes eight different genotypes (HEV-1 to 8) with five genotypes (HEV-1, 2, 3, 4 and 7) infecting humans [7,8]. All HEV genotypes belong to a single serotype [9]. The HEV-1 and HEV-2 genotypes are highly endemic in developing regions and are diffused through contaminated water. By contrast, genotypes 3 and 4 affect both animals (particularly swine) and humans and are the predominant HEV strains in several developed countries [3].

HEV-3 and -4 genotypes are thought to be transmitted through foods, mainly via the consumption of undercooked or raw pig, wild boar and deer meat [5]. Domestic swine and wild boars appear to represent the principal source of zoonotic HEV transmission in Europe and are regarded as the main viral reservoir [5,10].

Wild boar is one of the most common and widespread wildlife species worldwide [11]. A recent report has estimated in Italy a wild boar population of a minimum of 1.5 million animals in the year 2021, with about 300,000 wild boar heads killed per year in the period 2015–2021 [12].

The virus is mainly excreted through the fecal-oral route in wild boars/pigs, leading to the accumulation and persistence of HEV in the environment. Thus, HEV transmission might be influenced by contact between individuals and by environmental exposure [5]. The European Food Safety Authority (EFSA) has estimated that consumption of meat from infected animals or food products contaminated with HEV from human or animal origin represents an important public health risk. The One Health approach is pivotal to limiting HEV transmission [5].

The role of wild animals, and in particular wild boar, as a source of HEV in foodborne transmission, has been increasingly recognized. The consumption of raw wild boar meat and liver could represent a risk, as well as contact with wild boars, including hunting and slaughtering [9]. Moreover, given the high seroprevalence rates observed in swine workers, veterinarians, farmers, hunters and abattoir workers, occupational exposure surely plays a significant role [13].

Several epidemiological studies conducted in wild boar populations across European countries, including Italy, reported HEV RNA prevalence rates ranging from 0% to 56.3% and seroprevalence rates ranging from 4.8% to 58.9%. [14].

Information regarding the prevalence of HEV in the wild boar population in Southern Italy is limited, and surveys have not been conducted in the Apulia and Basilicata regions. In particular, most studies have been conducted in Central Italy (Abruzzo, Lazio and Umbria regions), where high numbers of HEV human cases and higher seroprevalence rates among blood donors have been reported [6,15]. However, a 2021 investigation detected HEV in shellfish produced from harvesting areas located in the Apulia region [16], suggesting the existence of a local ecological cycle for HEV. The presence of HEV in shellfish may indeed imply the pollution of the shellfish harvesting area by swine and wild boar waste and/or by urban sewage.

The aim of this study was to investigate HEV prevalence and gather information on the genetic diversity of HEV in wild boars hunted for domestic consumption in the Apulia and Basilicata regions. Also, since wild boar may enter into contact with pigs in rural settings with semi-intensive and free-range pig farming, this information could be useful to assess the risk of transmission of HEV from wild boar to farmed pigs.

## 2. Materials and Methods

### 2.1. Sampling

A total of 125 wild boar (*Sus scrofa*) samples (78 livers and 47 muscles) were collected in 2021 in the Apulia and Basilicata regions during the regular hunting season. In particular, 50 samples (47 muscles and 3 livers) were collected from different prefectures (Foggia, Barletta Andria Trani, Bari) in the Apulia region and 75 liver samples in the prefecture of Potenza in Basilicata. Each sample was obtained from a single animal. Gender, age, weight, location, and the date of hunting were recorded for each animal. According to the regional hunting calendars, samples were collected in January, November and December in Apulia and between April and July in Basilicata. The animals were classified as juveniles (<12 months of age), subadults (>12 months and <24 months) and adults (>24 months), based on tooth eruption patterns. All the collected samples were transported to the laboratories (Istituto Zooprofilattico Sperimentale della Puglia e della Basilicata, Foggia, Italy) under refrigerated conditions and stored at 4 °C until analysis.

### 2.2. Sample Preparation and Nucleic Acids Extraction

The presence of HEV was determined as previously described [17] using quality assurance control based on the standardized ISO 15216-2:2019 method [18]. Briefly, sample preparation was performed, as previously described [19], with slight modifications. In detail, 5 g of the sample was blended and transferred to a stomacher bag and spiked with 10 µL of a suspension of mengovirus (strain MC0, 1.6 × 10^5^ TCID50/mL), which was used as sample process control. Seven milliliters of TRIzol^TM^ reagents (Invitrogen ThermoFisher Scientific, Frederick, MD, USA) were added, and samples were homogenized at a maximum speed for 2 min. The liquid phase was recovered, transferred into a clean tube and clarified by centrifugation at 10,000× *g* for 20 min at 4 °C. After centrifugation, 1.4 mL of chloroform (0.2 *v*/*v*) was added to the supernatant, vortexed for 15 s, and then incubated at room temperature for 15 min. Thereafter, the samples were again centrifugated at 10,000× *g* for 15 min at 4 °C; the aqueous phase was retained, and its volume was measured. The suspensions were stored at −80 °C until nucleic acid extraction. Viral nucleic acids were extracted from 1 mL of the samples’ suspensions using the NucliSens MiniMag extraction system (bioMerieux, Paris, France) according to the manufacturer’s protocol, and the eluted RNA (100 µL) was immediately analyzed or stocked at −80 °C until use.

### 2.3. Real-Time Quantitative Reverse Transcription PCR (RTqPCR)

Real-Time Quantitative Reverse Transcription PCR (RTqPCR) for HEV was carried out on a CFX96 Real-Time PCR system thermocycler (Bio-Rad, Hercules, CA, USA) using the RNA UltraSense^TM^ One-Step qRT-PCR System kit (Invitrogen, Carlsbad, CA, USA) and primers and probe targeting the ORF3 region of HEV genome (expected amplicon size 69 bp) [20,21].

The thermal cycling conditions were 50 °C for 60 min and 95 °C for 5 min, followed by 45 cycles of 95 °C for 15 s, 60 °C for 1 min and 65 °C for 1 min. An external amplification control (in vitro synthesized RNA supplied by the Istituto Superiore of Sanità, Rome, Italy) was added to the samples to evaluate the presence of PCR inhibitors. Positive HEV samples were quantified, according to Di Pasquale et al. [17], with the results defined as number of HEV genome copies (g.c.) per gram of tissue, and calculated on the basis of the standardized ISO 15216-1:2017 method [22]. In detail, quantification was performed using a linearized plasmid containing the target sequence of HEV to generate the standard curve. Analyses were performed in duplicate, and the average concentration of the two replicate reactions was used for quantification.

### 2.4. RT-PCR and Sequence Analysis

Samples testing positive via RT-qPCR were subjected to HEV genotyping by nested RT-PCR amplification of a portion of the ORF1 and ORF2 regions [23]. The obtained nested PCR products were visualized by gel electrophoresis (1.5% agarose gel) using SYBR^®^ Safe DNA gel staining in 0.5X Tris-borate-EDTA (TBE) buffer (Invitrogen, Carlsbad, CA, USA). The amplicons were excised from the gel and purified using the commercial extraction kit QIAquick Gel Extraction Kit (Qiagen, Hilden, Germany) and were subjected to direct automated sequencing on both strands (BMR Genomics, Padova, Italy). Raw sequences were edited using the CLC Main Workbench software version 8.1 (QIAGEN, Aarhus, Denmark). The web-based Basic Local Alignment Search Tool (BLAST; http://www.ncbi.nlm.nih.gov, accessed on 20 March 2023) was used with default settings to find homologous hits. For genotyping, the consensus sequences were submitted to the HEVnet Typing Tool (http://www.rivm.nl/mpf/typingtool/hev/, accessed on 20 March 2023) [24].

### 2.5. Full-Length Genome Sequencing of HEV

The samples genotyped via nested RT-PCR underwent a PCR amplification of larger segments to obtain full-length genome sequences. The whole genome of HEV was reconstructed using an RT-PCR strategy with two overlapping large genome fragments. A 4.6 kb cDNA genomic fragment at the 5′ end and a 3.2 kb cDNA genomic fragment at the 3′ end were synthesized using SuperScript III First-Strand Synthesis SuperMix and reverse primers 4598 Rev [25] and QT [26], respectively. The genomic fragment at the 5′ end was amplified using a combination of the forward primer ORF1F [23] and reverse primer 4598 Rev [23] while, for the genomic fragment at the 3′ end, a forward primer 4228 FW [24] and reverse primer QT [26] were used. Long PCRs were carried out using the LA PCR Kit (version r.2.1) (Takara Bio, Tokyo, Japan) in a 50 µL reaction containing 1 mmol/L of primers, LA PCR Buffer (Mg^2+^), 8 µL of the dNTP mixture (corresponding to 400 mmol/L of each dNTP), 2.5 units of TaKaRa LA Taq polymerase and 1 µL of template DNA. The thermal protocol included an initial denaturation at 94 °C for 2 min, followed by denaturation at 94° for 30 s, annealing at 42 °C for 30 s and extension at 68 °C for 20 min; subsequently, a total of 40 cycles of denaturation at 94° for 30 s, annealing at 48 °C for 30 s and extension at 68 °C for 5min, followed by a final extension at 68 °C for 10 min were performed.

PCR products were subjected to electrophoresis on 1.5% agarose gel containing a fluorescent nucleic acid marker (GelRed; Bio-Rad Laboratories, Hercules, CA, USA) at 80 V for 45 min, and visualized under fluorescent light on the Gel Doc EZ Imaging System with Image Laboratory Software version 6.1 (Bio-Rad Laboratories).

PCR products were obtained for isolate ITA/2021/wild boar/337 and, for full genome sequencing, were quantified by the Qubit dsDNA HS assay (Thermo Fisher Scientific, Waltham, MA, USA) and used for library preparation with Genomic DNA by Ligation kit SQK-LSK110 (Oxford Nanopore Technologies, ONT, Oxford, UK) following the manufacturer’s guidelines. Libraries purification was carried out using Agencourt AMPure XP magnetic beads (Beckman Coulter™, Brea, CA, USA) and sequencing with flowcell flongle FLO-FLG001, R9.4.1 version adapted in a MinION Mk1C (ONT, Oxford, UK) platform for 24 h. FastQ MinION files were subjected to quality control, trimming and reference assembly by Minimap2 and open reading frame (ORF) predictions, and annotations were performed in Geneious Prime software v. 2021.2.2 (Biomatters Ltd., Auckland, New Zealand).

### 2.6. Phylogenetic Analysis

Sequence editing and multiple alignments were performed using Geneious Prime version 2021.2 (Biomatters Ltd., Auckland, New Zealand). The sequences were aligned with genetically related HEV strains retrieved from the GenBank database by MAFFT [27]. The correct substitution model for phylogeny was obtained using MEGA X version 10.0.5 software [28]. The evolutionary history was inferred using the maximum-likelihood method, the Tamura-Nei 4-parameter model, a discrete gamma distribution and invariant sites to model evolutionary rate differences among sites (6 categories) and supply statistical support with 1000 replicates. Bayesian inference and neighbor-joining phylogenetic approaches were explored.

### 2.7. GenBank Sequence Submission

The complete genome sequence of strain ITA/2021/wild boar/337 was deposited in the GenBank database under accession number OQ286030.

### 2.8. Statistical Analysis

The prevalence of HEV was calculated with the 95% confidence interval (CI) using the modified Wald method. The Chi-square test with Yates’ correction was executed to compare the significance (*p*-value) of differences between positive and negative results from the sampling region, including muscle and liver samples, and from gender. All statistical calculations were performed using the GraphPad Software QuickCalcs (https://www.graphpad.com/quickcalcs/ accessed on 3 April 2023).

## 3. Results

### 3.1. Molecular Detection and Quantification of HEV RNA

Overall, out of 125 wild boar samples collected in this study, HEV RNA was detected in 13 samples (10.4%, 95% CI: 6.06–17.11%) with Cq values ranging from 21 to 41. In detail, 6 (12%) out of 50 and 7 (9.3%) out of 75 samples from the Apulia and Basilicata regions, respectively, tested positive for HEV using RT-qPCR. Moreover, 6 (12.8%) out of 47 muscle samples and 7 (9.0%) out of 78 liver samples were positive for HEV (Table 1).

No statistically significant difference was detected in terms of the prevalence of HEV in wild boars based on their tissue type (*p* = 0.7112), including the geographic location of sampling (*p* = 0.8576) and gender (*p* = 0.3687).

As for the quantitative data, 9 samples (69.2% of the positive samples) showed HEV levels ≥ limit of quantification (LOQ) (1.4 g. c./µL ≡ 3.2 × 10^2^ g.c./g, according to published data [15]) whilst 4 samples tested positive at levels below the limit of quantification of the method (Table 1). The viral loads differed between these two regions. In the Apulian samples, HEV viral loads were higher than in samples from Basilicata (Table 1).

### 3.2. Genotyping and Phylogenetic Analysis

The 13 samples that were positive for HEV in RT-qPCR were subjected to RT-PCR amplification of the partial ORF1 and ORF2 regions. Seven samples (7/13; 53.8%) tested positive for both RT-PCR protocols and were subjected to direct sequencing. Attempts to genotype the other six positive samples were unsuccessful, as conventional nested RT-PCR amplification yielded negative results. This could be related to the low concentration of HEV in the samples (Cq values > 37). As for the seven genotyped samples, four HEV strains showed the highest nt identity (94.9–96.2%) in the ORF2 to the HEV-3c strain HEVCU634 (MH450021). One such strain (ITA/2021/wild boar/37155-1) displayed a high identity (96.5% nt) to strain HEVCU634 (MH450021) in the ORF1 region (Table 2). Strain ITA/2021/wild boar/287 displayed the highest nt identity to the HEV-3c strain wbGER27 in the ORF1 (98.8%) and to strain HEV-3c S03_Germany (MT497761) in the ORF2 (95.9%). Strain ITA/2021/wild boar/C1 displayed the highest nt identity in both ORF1 (94.2%) and ORF2 (95.1%) regions to the HEV-3f2 strain 179535MM66 (GenBank accession nr MZ289122), whilst strain ITA/2021/wild boar/337 shared the highest nt identity with both ORF1 (98.3%) and ORF2 (90.9%) regions to the HEV 3m strain HESQL050 (MW355399) (Table 2). Using the HEVnet typing tool, partial ORF2 sequences of the HEV strains were characterized as genotype 3. Three different subtypes were identified, i.e., 3c (n = 5), 3f (n = 1) and 3m (n = 1) (Table 2).

**Figure 1:**
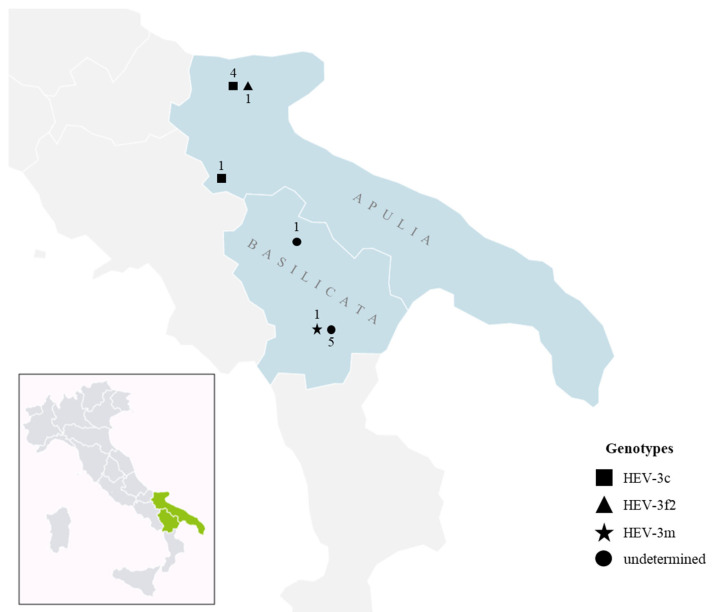
Geographic distribution of the different subtypes of hepatitis E virus strains in wild boar from the Apulia and Basilicata regions.

Figure 1 displays the geographic distribution in Apulia and Basilicata regions of the HEV positive samples characterized at the genotype and subtype level.

**Figure 2:**
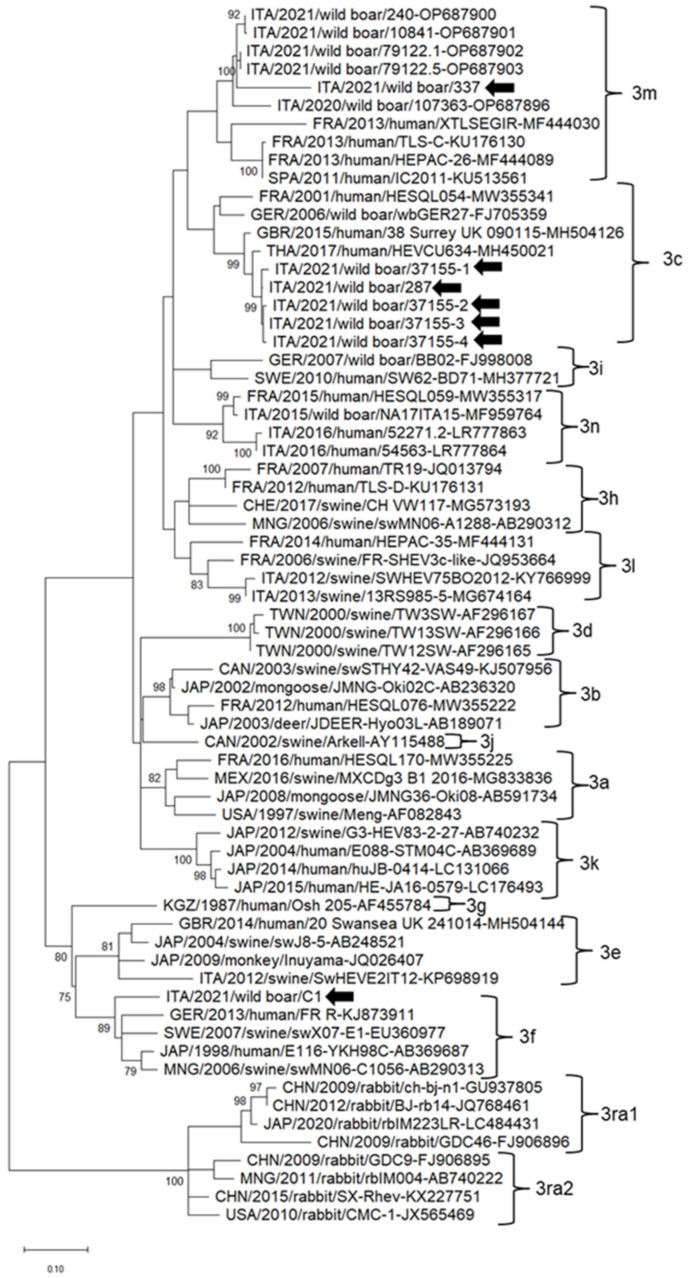
Partial ORF2-based unrooted phylogenetic tree of hepatitis E virus strains identified in this study and reference strains recovered in the GenBank database. The Maximum Likelihood method and Tamura–Nei model (four parameters) with gamma distribution and invariable sites were used for the phylogeny. A total of 1000 bootstrap replicates were used to estimate the robustness of individual nodes on the phylogenetic tree. Bootstrap values greater than 75% were indicated. Black arrows indicate strains detected in this study. The scale bar indicates nucleotide substitutions.

In the phylogenetic tree based on the partial ORF2 region, the HEV strains identified in this study clustered into three different clades together with the strains retrieved worldwide. The HEV strains ITA/2021/wild boar/37155-1, ITA/2021/wild boar/37155-2, ITA/2021/wild boar/37155-3 and ITA/2021/wild boar/37155-4 belonged to the clade 3c along with HEV strains detected in European and Asian countries. HEV strain ITA/2021/wild boar/C1 clustered with HEV strains identified in Europe and Asia within clade 3f. HEV strain ITA/2021/wild boar/337 clustered within subtype 3m together with HEV strains detected in wild boars in Italy and in human patients in France and Spain (Figure 2).

The comparison of the maximum likelihood phylogeny with Bayesian inference and neighbor-joining approaches demonstrated similar topologies with slight differences in bootstrap values at the nodes of the tree. Accordingly, the maximum likelihood tree was retained.

## 4. Discussion

Recently, we reported the detection of HEV RNA in 2 (0.89%) out of 225 shellfish samples collected from a harvesting area in the Apulia region [16]. This suggested the existence of a local ecological cycle for HEV, although the origin of this viral contamination could not be determined. The presence of HEV in shellfish could result from contamination with swine and wild boar waste and/or urban sewage, as previously investigated [29]. This provided a rationale to investigate the presence of HEV in wild boar.

The results of the present study confirm the presence of HEV in samples from wild boar hunted for domestic consumption in Apulia and Basilicata regions, even if a lower prevalence was found compared to other Italian areas. The prevalence of HEV in wild boar likely differs significantly among Italian regions due to the different types of samples collected, different sampling strategies, and the different sensitivity and specificity of diagnostic protocols [17,30,31,32,33,34,35,36,37,38,39,40,41,42,43,44,45,46,47]. The prevalence of HEV RNA herein reported is similar to that from nearby regions of central Italy, as reported in Abruzzo (9.5%) [47], Lazio (12.1%) and Campania (7.5%) [39].

Quantitative data are also pivotal in order to define the risk related to the consumption of wild boar products, particularly for traditional regional dishes such as dried sausages of wild boar meat. Genetic differences among wild boar sub-populations could determine changes in the patterns of HEV infection and also, therefore, in viral loads across different organs/tissues. The mean value reported in the present study (2.32 × 10^4^ g.c./g) was similar to the mean value reported in another Italian study (1.85 × 10^4^ g.c./g.) in Central Italy [17]. Moreover, these findings are similar to those reported by similar studies conducted in European countries [48,49,50]. Most Italian studies have investigated the presence of HEV in liver tissues, and only a few studies have analyzed muscle tissues, with a prevalence rate of 55% [39] and 1.35% [45]. As shown in Table 1, in the muscle samples, we detected viral loads higher than in liver samples and also at higher prevalence rates. This is in agreement with the study by De Sabato et al. [39]. Although the viral load in the liver is usually higher compared to muscle or meat [51], it has been shown in experimentally inoculated pigs that the presence of HEV in meat depends on the time of infection, and it is more probable in late stages of infection, suggesting a hypothetical replication of HEV in other anatomical parts [52]. Moreover, the presence of HEV in muscles could be due to a viraemic status [53]. In other studies, in domestic pigs, the detection of HEV in the liver was not associated with the presence of the virus in the muscle tissues (e.g., loin and ham), probably due to a low viral load under the limit of detection of the applied method [54,55]. Moreover, Salines et al. [56] hypothesized that under certain circumstances (e.g., coinfection with other viruses), HEV could replicate even in muscles, leading to a higher risk of contaminated meat due to asymptomatic infection with HEV.

Epidemiological studies in Italy have shown that subtype 3f is the most common subtype detected in humans and animals, followed by subtype 3c [57,58]. The same scenario has been reported in humans in other European countries [51,59]. Interestingly, after 2010, in some European countries, a shift in subtypes was observed among HEV-3 human strains, and the subtype HEV-3c became predominant in France, England, Wales, The Netherlands, Germany, and since 2016, in Belgium [51,59,60,61]. In Italy, this shift was not observed. Certainly, HEV-3f was the first ever detected subtype (the year 1985) in Italy among swine and wild boar, whilst subtype 3c was identified from 2006 onwards [57]. The shift in HEV-3 subtypes in Europe could be attributed to the trading of pigs in European countries. The subtype HEV-3c might have been introduced to Italy with the trading of meat or live animals from Central European countries [57,58].

In the present study, the full-length genome sequence of a HEV-3m strain from the Basilicata region was obtained. This subtype 3m (previously named 3chi-new) [62] was first detected in a Spanish patient in 2011 [63] but likely circulated in wild boar in Spain as early as 2010 [64], and it is now regarded as the second most common HEV subtype in Spanish patients [65]. The subtype HEV-3m has since then been detected in human patients in France, Belgium, The Netherlands, the United Kingdom [61,62,66], and recently in Sweden [67], suggesting that it is zoonotically transmitted by contaminated meat or water or direct contact with wild boars [68]. The subtype 3m was also identified in Italy in 2022 from wild boar of the Liguria region (North Italy) [46], although only partial sequences were generated from the virus. Our findings suggest that the subtype HEV-3m is not restricted to Northern Italian areas. The circulation of different subtypes and/or the emergence of new subtypes could have implications for human health, determining changes in the patterns of HEV infection and disease in humans. Even if several studies have shown no significant association between the HEV subtype and the severity of symptoms [62,69], it has been estimated to have a lower risk of hospitalization for patients infected with subtype HEV-3c than for patients infected with subtype HEV-3f or HEV-3e [70,71].

Moreover, the role of domestic pigs as a major risk of HEV transmission to humans should not be overlooked. An Italian national project investigated the presence of HEV in pigs from four abattoirs located across the country between 2017 and 2019 [72]. HEV RNA was detected in 3.6% of the samples, and 76.8% of sera and meat juice were positive for the presence of anti-HEV antibodies. The identified strains belonged to an unclassified subtype of HEV-3 and subtype 3c. Recently, HEV RNA was detected in 17.5% of pigs slaughtered in the Sicily region, with most of these strains belonging to subtype 3c [73]. Interestingly, the same strains were identified in samples from Calabrian wild boars, suggesting a possible direct transmission between wild boars and pigs. Further studies should be addressed to investigate the environmental interaction between wild boar and domestic pigs.

## 5. Conclusions

This study provides evidence of the presence of HEV RNA in samples from wild boar hunted for domestic consumption in Southern Italy, in two regions (Apulia and Basilicata) with peculiar agricultural and zootechnic features that are markedly different from Northern regions. The emerging subtype HEV-3m was identified in Southern Italy, hinting at the circulation of this subtype in local wild boar populations. The high prevalence and viral loads of HEV RNA in wild boar meat samples highlight an important health risk associated with the consumption of food based on raw meat. Gathering information on the HEV strains circulating in different geographical areas is useful for tracking the origin of HEV cases in human patients and assessing the epidemiological role of wild boar as a potential virus reservoir for farmed pigs.

## Figures and Tables

**Table 1:** Prevalence and viral load of HEV detected in wild boar samples. Each sample was obtained from a single animal.

	N. of Tested Samples	N. of Positive Samples (%)	Viral Load		Geometric Mean of Quantifiable Samples (g.c./g)
			<LOQ	≥LOQ	
**Region**					
Apulia	50	6 (12.0%)	-	6	1.50 × 10^5^
Basilicata	75	7 (9.3%)	4	3	2.81 × 10^4^
**Sample Type**					
Muscle	47	6 (12.8%)	-	6	1.5 × 10^5^
Liver	78	7 (9.0%)	4	3	2.81 × 10^4^
**Gender**					
Male	48	3 (6.2%)	2	1	4.82 × 10^4^
Female	77	10 (12.9%)	2	8	9.21 × 10^4^
**Age (months)**					
<12	10	-	-	-	-
13–24	43	6 (13.3%)	1	5	2.06 × 10^5^
>24	72	7 (9.7%)	3	4	2.86 × 10^4^
Total	125	13 (10.4%)	4	9	6.49 × 10^4^

**Table 2:** Results of data analysis on the sequences generated in this study. The source, year of collection and the best match using the Blast Nucleotide interrogation tool from the National Center for Biotechnology Information database (accessed on 27 March 2023) are shown. Classification at the genotype and subtype level is based on NCBI taxonomical and HEVnet typing tool assignments.

Sample	Hevnet Assignment		Identity in the NCBI Database					
			ORF1			ORF2		
	Genotype	Subtype	Accession nr	Reference Strain	nt Identity %	Accession nr	Reference Strain	nt Identity %
ITA/2021/wild boar/C1	3	f	MZ289122	isolate 179535MM66	94.2	MZ289122	isolate 179535MM66	95.1
ITA/2021/wild boar/287	3	c	FJ705359	isolate wbGER27	98.8	MT497761	isolate S03_Germany	95.9
ITA/2021/wild boar/37155-1	3	c	MH450021	isolate HEVCU634	96.5	MH450021	isolate HEVCU634	94.9
ITA/2021/wild boar/37155-2	3	c	MF444063	isolate HEPAC-11	97.1	MH450021	isolate HEVCU634	95.7
ITA/2021/wild boar/37155-3	3	c	MH504129	isolate HEV 35_Poole_UK_050115	98.8	MH450021	isolate HEVCU634	96.2
ITA/2021/wild boar/37155-4	3	c	MH504129	isolate HEV 35_Poole_UK_050115	96.5	MH450021	isolate HEVCU634	95.7
ITA/2021/wild boar/337 *	3	m	MW355399	isolate HESQL050	98.3	MW355399	isolate HESQL050	90.9

* Full-length genome available.

## Data Availability

The complete genome sequence of strain ITA/2021/wild boar/337 has been deposited in the GenBank database under accession number OQ286030.
